# Dose-Dependent Effects of Closed-Loop tACS Delivered During Slow-Wave Oscillations on Memory Consolidation

**DOI:** 10.3389/fnins.2018.00867

**Published:** 2018-11-27

**Authors:** Aaron P. Jones, Jaehoon Choe, Natalie B. Bryant, Charles S. H. Robinson, Nicholas A. Ketz, Steven W. Skorheim, Angela Combs, Melanie L. Lamphere, Bradley Robert, Hope A. Gill, Melissa D. Heinrich, Michael D. Howard, Vincent P. Clark, Praveen K. Pilly

**Affiliations:** ^1^Psychology Clinical Neuroscience Center, The University of New Mexico, Albuquerque, NM, United States; ^2^Department of Psychology, The University of New Mexico, Albuquerque, NM, United States; ^3^Center for Human-Machine Collaboration, Information and Systems Sciences Laboratory, HRL Laboratories, LLC, Malibu, CA, United States; ^4^The Mind Research Network, Albuquerque, NM, United States; ^5^Department of Neuroscience, University of New Mexico School of Medicine, Albuquerque, NM, United States

**Keywords:** memory consolidation, closed-loop, tACS, sleep, tDCS, learning and memory

## Abstract

Sleep is critically important to consolidate information learned throughout the day. Slow-wave sleep (SWS) serves to consolidate declarative memories, a process previously modulated with open-loop non-invasive electrical stimulation, though not always effectively. These failures to replicate could be explained by the fact that stimulation has only been performed in open-loop, as opposed to closed-loop where phase and frequency of the endogenous slow-wave oscillations (SWOs) are matched for optimal timing. The current study investigated the effects of closed-loop transcranial Alternating Current Stimulation (tACS) targeting SWOs during sleep on memory consolidation. 21 participants took part in a three-night, counterbalanced, randomized, single-blind, within-subjects study, investigating performance changes (correct rate and F1 score) on images in a target detection task over 24 h. During sleep, 1.5 mA closed-loop tACS was delivered in phase over electrodes at F3 and F4 and 180° out of phase over electrodes at bilateral mastoids at the frequency (range 0.5–1.2 Hz) and phase of ongoing SWOs for a duration of 5 cycles in each discrete event throughout the night. Data were analyzed in a repeated measures ANOVA framework, and results show that verum stimulation improved post-sleep performance specifically on generalized versions of images used in training at both morning and afternoon tests compared to sham, suggesting the facilitation of schematization of information, but not of rote, veridical recall. We also found a surprising inverted U-shaped dose effect of sleep tACS, which is interpreted in terms of tACS-induced faciliatory and subsequent refractory dynamics of SWO power in scalp EEG. This is the first study showing a selective modulation of long-term memory generalization using a novel closed-loop tACS approach, which holds great potential for both healthy and neuropsychiatric populations.

## Introduction

Sleep is hypothesized to promote the consolidation of information from short-term stores to more schematized long-term representations (Fenn et al., 2003; Stickgold and Walker, 2013; Sterpenich et al., 2014; Friedrich et al., 2015). Slow-wave oscillations (SWOs) are a global neural phenomenon exhibiting synchronized EEG activity at a frequency peaking at 0.7–0.8 Hz, which appear to be involved in declarative memory consolidation (Plihal and Born, 1997; Marshall and Born, 2007; Diekelmann and Born, 2010; Rasch and Born, 2013). They are thought to promote large-scale neuronal synchronization across cortical and sub-cortical regions (Timofeev et al., 2012), which is ideal for coordinated reactivations (or replays) across disparate brain regions, facilitating the transfer of information from hippocampal to neocortical areas (Squire and Alvarez, 1995). Nested within these SWOs are sleep spindles and ripples, which are thought to help coordinate this transfer (Oudiette and Paller, 2013). Coordinated hippocampal and cortical replays (Ji and Wilson, 2007) may also be involved in building cognitive schemata by selectively strengthening shared elements across multiple episodes through complementary learning systems (CLS) processes (McClellend et al., 1995; Kumaran et al., 2016). Alternately, synaptic homeostasis mechanisms during sleep, which are augmented by SWOs, may lead to a more generalized, consolidated representation of information acquired during waking (Tononi and Cirelli, 2003, 2006). Sleep-dependent consolidation effects have been revealed in innumerable studies with various forms of information, including extraction of the hidden structure of digit strings (Wagner et al., 2004) and Serial Reaction Time Task patterns (Fischer et al., 2006), as well as incorporation of new words into an existing vocabulary (Tamminen et al., 2010).

The use of non-invasive transcranial electrical stimulation (tES) that mimics some aspects of these SWOs has been shown to improve memory performance in previous studies, by potentially modulating the above-mentioned sleep consolidation processes. Marshall et al. (2004) administered 0.26 mA/cm^2^ anodal transcranial direct current stimulation (tDCS) over bilateral frontal electrode sites during wake and separately during SWO-rich sleep stages repeatedly for 30 min in 15-s on/off cycles, thus manually approximating a sawtooth-like waveform. Declarative memory retention following sleep was improved in the verum condition only when stimulation was delivered during sleep compared to wake or placebo administration, suggesting that sleep-dependent memory processes were being modulated. Subsequently, Marshall et al. (2006) applied 0.75 Hz non-negative transcranial Alternating Current Stimulation (tACS), which is called offset oscillatory tDCS, at a maximum current density of 0.517 mA/cm^2^ over bilateral frontolateral locations and mastoids starting 4 min after participants had entered Non-Rapid Eye Movement (NREM) stage 2 sleep. Stimulation was applied for 5 five-min intervals with a 1-min break in between intervals. This open-loop offset oscillatory tDCS protocol led to a better overnight performance change for verum stimulation compared to sham in a verbal paired associates task (PAT), where words are studied in pairs during training, followed by tests where a cue word is presented alone and the paired word must be recalled. Westerberg et al. (2015) delivered offset oscillatory tDCS over F7, F8, and at mastoids with stimulation parameters of Marshall et al. (2006) on older adults during slow-wave sleep (SWS) in a 90-min nap. They also showed that the amplitude of SWOs during the stimulation-free intervals was higher in the experimental group, who also showed a larger improvement in word-pair recall compared to the sham group. A recent study by Ladenenbauer et al. (2016) also showed a performance improvement in visual declarative memory using a similar offset oscillatory tDCS protocol in older adults with mild cognitive impairment during a daytime nap compared to sham, with a concomitant enhancement of SWOs and SWO-spindle coupling.

However, these findings are not without controversy and have not always been replicated. For example, Eggert et al. (2013) failed to show a benefit of SWO augmentation on declarative memory assessed via a PAT in 26 elderly adults using the stimulation protocol outlined in Marshall et al. (2006), which is in line with another study in older healthy adults (Paßmann et al., 2016). Sahlem et al. (2015) also failed to find a declarative memory benefit using a protocol similar to Marshall et al. (2006) with an offset oscillatory square waveform. One possibility of the inconsistency across studies is that stimulation was delivered open-loop, without considering the phase and frequency of ongoing neural activity, as opposed to closed-loop, where the neural oscillations are sensed, and the stimulation is applied synchronously. Closed-loop stimulation has been shown to be more effective than open-loop control in a computational neural model of Parkinson’s disease (Li et al., 2015), and has been argued to have the potential for modulating brain activations in a state-dependent manner for performance optimization in various tasks (Zrenner et al., 2016).

There is considerable experimental (Marshall et al., 2006, 2011; Herrmann et al., 2013; Helfrich et al., 2014) and computational (Ali et al., 2013; Merlet et al., 2013) evidence that tACS can effectively entrain brain oscillations, and that the phase at which tACS is delivered to the cortex is critical to drive entrainment (Ali et al., 2013). For instance, Helfrich et al. (2014) applied 1.0 mA tACS at 10 Hz over parieto-occipital cortex while simultaneously recording EEG during a visual oddball task. To investigate phase-dependent effects, the visual stimulus was presented at four different phases of the tACS waveform. The data demonstrated a significant enhancement in alpha band (8–12 Hz) power during verum stimulation, as well as an interaction between stimulation condition and phase of stimulus presentation on target detection, suggesting that tACS modulated performance in a phase-dependent manner.

Computational modeling work corroborates the importance of phase for oscillatory entrainment with tACS (Ali et al., 2013). Here, a simulated neural network of 200,000 neurons (160,000 pyramidal cells and 40,000 interneurons) was subjected to either a direct current waveform or an alternating current waveform at 3 Hz (the endogenous frequency of the network). The simulated tACS current produced larger spectral power at 3 Hz in network activity, measured by local field potential, compared to transcranial Direct Current Stimulation (tDCS). Although tACS could successfully entrain the network oscillations regardless of onset phase, phase played an important role in the speed at which networks were entrained. Stimulation starting near the hyperpolarizing DOWN state of the endogenous oscillation (close to π) reset the excitatory synapses, allowing for greater excitatory activity in the network during the subsequent depolarizing UP state, leading to faster entrainment time and greater synchronization. These experimental and computational results suggest that tACS is a better candidate than tDCS to entrain neurons and network activity, and the phase at which tACS is applied is crucial.

Several studies have employed closed-loop auditory stimulation delivered at predicted UP states during sleep to enhance the SWOs (Ngo et al., 2013a,b; Cox et al., 2014; Ong et al., 2016; Papalambros et al., 2017). Closed-loop tACS may provide an alternative and potentially a more effective method for modulating the distributed synchronous phenomenon underlying SWOs. In particular, tACS utilizes currents with full oscillatory dynamics to directly modulate these brain oscillations. Furthermore, being non-sensory, tACS is not influenced by ambient sensory stimuli in the environment during sleep. Any closed-loop stimulation method is technically complex, and the electrical versions have only recently been demonstrated (Wilde et al., 2015). Lustenberger et al. (2016) used feedback-controlled tACS at 12 Hz that was triggered by ongoing spindle activity during sleep to modulate motor, but not declarative, memory consolidation, but no one has yet developed a method to deliver tACS during SWOs in a closed-loop fashion to investigate hippocampally-dependent memory consolidation. Given the inconsistent findings of using tES to augment SWOs and memory performance, as well as the fact that no study to date has investigated the effects of phase- and frequency-locked non-invasive stimulation, we tested the effects of closed-loop tACS during sleep on performance in a target detection task that has been shown to be sensitive to the effects of waking tDCS during training (Clark et al., 2012; Coffman et al., 2012; Falcone et al., 2012). In this study and Ketz et al. (2018), we investigated the effects of combining tDCS during waking training and closed-loop tACS during slow-wave oscillations on memory performance assessed over 24 h. In particular, we assessed the behavioral effects of this intervention on veridical versus generalized forms of memory (Coffman et al., 2012) in pre-sleep and post-sleep tests, in order to differentiate the schematization of learned information from rote recall (Stickgold and Walker, 2013). The intention was to produce the largest total improvement in learning possible, thus a combination of electrical augmentation techniques, one during training and another overnight, was utilized. The unique contribution of each technique was not investigated here. Ketz et al. (2018) analyzed overall sleep EEG biomarkers induced by our closed-loop stimulation in the context of well-recognized NREM EEG measures related to memory consolidation. The current study presents a more thorough analysis of the behavioral effects with two different metrics (namely, correct rate and F1 score) and over the morning and afternoon post-sleep tests separately, including data from five additional subjects who could not be included for the biomarker analyses in Ketz et al. (2018). Furthermore, the current study analyzes the explicit dose effects of the number of transient closed-loop tACS applications through the night not only on the post-sleep behavioral performances, but also on the post-tACS changes of SWO power in scalp EEG during sleep. It also discusses the implications of these findings for optimizing the behavioral efficacy of our novel closed-loop slow-wave tACS intervention for future performance augmentation applications.

## Materials and Methods

Note the descriptions of the experimental methods below have also been presented in Ketz et al. (2018).

### Inclusion/Exclusion Criteria

Participants were 18–40 years of age, used English as a first language, completed high school, and had no history of head injury with loss of consciousness for longer than 5 min. They were right-handed according to the Edinburgh Handedness Inventory (Oldfield, 1971), had no history of neurological or psychiatric disorder, had no history of alcohol or drug abuse, were non-smoking, had no excessive alcohol or caffeine consumption, were not currently taking any medication significantly affecting the central nervous system, had no implanted metal, had no sensitivity or allergy to latex, had good or corrected hearing and vision, and reported no sleep disturbances. Women who were pregnant, or thought they may be, were also excluded.

### Participants

A total of 21 participants (mean age = 20.1 years, *SD* = 1.67 years, 8 female), including data from 5 additional subjects who could not be included for the biomarker analyses in Ketz et al. (2018) due to insufficient EEG data, were recruited using flyers placed around the campus of the University of New Mexico and surrounding community to complete both verum and sham stimulation conditions of this experiment and received monetary compensation. All participants provided signed informed consent to participate in the study in accordance with the Declaration of Helsinki, which was approved by the Chesapeake Institutional Review Board.

### Hidden Target Detection Paradigm/Experimental Procedure

**FIGURE 1:**
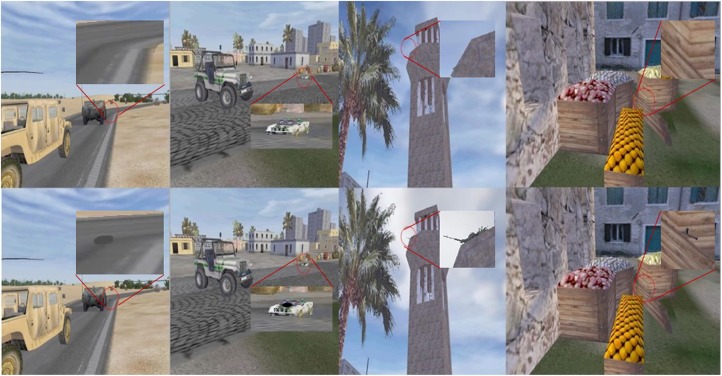
Example images used in the target detection paradigm. Top row images have no targets, while the corresponding images in the bottom row contain targets. First two columns show exemplar object target images, and last two columns show people target images. Red circles and inserts were added here to identify the targets and locations, but were not present in the images shown to participants.

A modified version of the original task, described in Clark et al. (2012), was created to allow for the within-subjects design of the current study. Participants were trained to discover the presence of targets hidden in complex static images, and changes in performance were tracked over time. Though not a traditional memory assay, this task was chosen in part because it could be used to examine different forms of memory encoding simultaneously, including both veridical memory and consolidated or generalized memory. As noted above, the aim of the current study was to produce as large a benefit for memory performance as possible, and thus the waking tDCS was leveraged in combination with sleep tACS to produce a synergistic, additive effect. Targets that were hidden in these images included explosive devices concealed by or disguised as dead animals (e.g., camels), roadside trash, fruit, flora, rocks, sand, or building structures; and enemies in the form of snipers, suicide bombers, tank drivers, or stone-throwers. The stimulus set was divided into two target categories: people targets (e.g., enemy snipers, friendly fire), and object targets (e.g., improvised explosive devices, trip wires). Half of the images presented to participants during tests following training were identical to those seen in training (repeated images, used to test veridical memory), and half were related, but with varying spatial perspective from the same corresponding scenes (generalized images, used to test for consolidated memory). Thus, this design allowed for the investigation of effects of the sleep intervention on both veridical recall and generalization performances. Examples of images presented to participants can be found in Figure 1. Participants were instructed that they could stop the task at any time if the stimuli were too uncomfortable or made them anxious. No participants elected to stop for such a reason.

The experiment was conducted over the course of 6 days that included three nights spent in our sleep laboratory referred to here as “acclimation,” “night 1,” and “night 2,” as well as two afternoon follow-up test sessions (“day 2 follow-up,” “day 3 follow-up”), as well as an initial orientation session. Participants were randomly assigned to one of four manipulations in a within-subjects, counterbalanced, single-blind design: Object Target/Sham Stimulation Day 2, People Target/Verum Stimulation Day 3 (SO/AP), Object Target/Verum Stimulation Day 2, People Target/Sham Stimulation Day 3 (AO/SP), People Target/Sham Stimulation Day 2, Object Target/Verum Stimulation Day 3 (SP/AO), People Target/Verum Stimulation Day 2, Object Target/Sham Stimulation Day 3 (AP/SO). The abbreviations indicate the condition on (day2/day3), where S = Sham, A = Verum, O = Object targets, and P = People targets. Stimulation conditions applied to both waking and sleep interventions (e.g., a “Verum” assignment meant that a full dose of both tDCS during wake and tACS during sleep were administered). This combined protocol was implemented to achieve as large a long-term effect on memory performance as possible, given that waking tDCS was previously shown to improve the immediate performance on the task that persisted over at least 24 h without any overnight changes (Falcone et al., 2012). At the orientation session, participants were invited to provide informed consent, and were given several questionnaires to assess various aspects of their personality and sleep habits, as well as to gather an IQ estimate. Following the questionnaires, head measurements were made (circumference, nasion to inion, and pre-auricular to pre-auricular) to fit an EEG cap. Participants were next given a tour of the sleep laboratories and an explanation of the EEG/Stimulation equipment and experimental procedures. Finally, each participant was issued a Fitbit wrist-worn biometric sensor (Dickinson et al., 2016) with instructions on how to correctly operate it to track sleep prior to their lab visits.

For the acclimation night, participants arrived at the sleep laboratory by 17:00, and were prepped and fitted with an EEG cap [see Waking Electroencephalographic (EEG) Data Collection section], and an adapted version of Raven’s Progressive Matrices called Sandia Matrices was administered (Matzen et al., 2010). Next, data was collected to calibrate biometrics (Patel et al., 2018) for use in a predictive computational model, including a breath count task to measure attentional lapses (Braboszcz and Delorme, 2011) that lasted 30 min, as well as a 3-back task to generate cognitive stress and mental fatigue (Hopstaken et al., 2015) that lasted 21 min was gathered. Participants could then relax in the laboratory until roughly 21:00, when they were prepped for PSG recording during sleep (see Polysomnographic Data Collection section). EEG electrode locations were digitized using Polhemus FASTRAK System (Polhemus, Inc.) for data analysis purposes as well as to measure how much the cap may have shifted during the subsequent sleep episode. Participants were instructed to lie down in a supine position at approximately 22:00, when biocalibrations were performed to help identify sources of noise in later EEG acquisition. This included EEG data collection of eyes open for 1 min, closed for 1 min, looking up, down, right, and left, blinking slowly 5 times, clenching the jaw, and finally moving into a comfortable sleeping position. Lights out for the participants occurred between 22:00–23:00, and they slept for up to 8 uninterrupted hours before being awoken. During sleep, EEG data were monitored, and the closed-loop prediction algorithm was started when 4 min of continuous N2/N3 sleep was observed by research assistants trained by a sleep research expert in identifying sleep stages based on PSG. During the acclimation sleep, no stimulation was applied, but the information gathered from the closed-loop prediction algorithm was used to verify the SWO relative power threshold of 20%, and reduced if needed, for subsequent experimental nights for each participant. Upon waking, participants could use the restroom and were offered water and snacks. They filled out the Karolinska Sleep Diary (KSD; Åkerstedt et al., 1994) to assess subjective sleep quality. Next, they completed a 1-back task for 21 min to assess alertness and were then disconnected and cleaned from the EEG hardware and released.

For night 1, participants arrived at the laboratory at approximately 17:30, and were immediately set up for EEG data collection and tDCS. Participants were seated in front of the computer and instructed on how to respond to the stimuli but were not given specific information about the nature of the hidden targets or any strategies with which to find them. First, participants performed two baseline runs (test blocks and two), consisting of 60 novel images per run. Each image was presented for 2 s, during which a binary response (target present/target absent) was made using the keyboard, and with an inter-image interval that varied from 4 to 8 s. Each baseline run lasted approximately 8 min, and no feedback was given regarding performance.

Participants then took a brief baseline mood questionnaire to help assess effects of tDCS on subjective mood. The mood questionnaire consisted of nine questions on a 0–5 Likert scale. Items included feelings of nervousness or excitement, tiredness, confusion, sadness, frustration, dizziness, nausea, physical pain or discomfort, and ability to pay attention. After all questions were answered, the training portion of the target detection task was administered.

Participants completed three training runs, the first two of which (training blocks one and two) were under 30 min of either verum (1.0 mA) or sham (0.1 mA) tDCS, followed by one more run (training block three) immediately following the administration of tDCS. The training blocks differed from the test blocks in that 1.5 s following each image presentation, the participants were given audiovisual feedback using a short clip (∼5 s) regarding the consequence of their decision. If the participant indicated “target present” and was correct, a short video depicting the mission progressing as planned was shown, with a voiceover praising the participant for choosing correctly. If the participant indicated that a target was present when there was not, a voiceover chastised them for delaying the mission, or insulted them by indicating they were acting cowardly. If the participant correctly indicated that there was no target present, feedback was given that the mission was progressing as planned. If they indicated that there was no target present when in fact there was one, a video showing the consequence of missing the target was shown. For example, another member of the participant’s platoon was shot by a sniper, or a Humvee was destroyed by an improvised explosive device. Further, a voiceover scolded the participant for missing the target and told them that members of their team had been killed. Each of the three training blocks consisted of 60 novel images and lasted approximately 16 min. The audiovisual feedback did not provide specific details of the shape or location of the target, but enough information was available from the test image and feedback movie that the participant could infer its type and general position in the image.

Following the three training runs, two more test runs (test blocks three and four; “immediate test”) were administered to gauge the immediate effect of tDCS on learning before sleep. Half of the stimuli used in the immediate test had been presented during training (repeated images), while the remaining stimuli were similar in content with the same targets seen before but had not been presented during training (generalized images). Each test block presented 60 images (30 repeated, 30 generalized). Thus, memory for trained images and the generalization of the training to novel images could be examined separately. Following the final test block, participants were administered an exit mood questionnaire consisting of the same nine questions in the initial mood assessment, as well as a questionnaire probing the strategy the participants used to complete the task. Next, a new set of Sandia Matrices was administered, as was a Language History Questionnaire (LHQ). Then participants could relax in the laboratory until roughly 21:00, when they were prepped for PSG recording during sleep (see Polysomnographic Data Collection section). EEG electrode locations were digitized, and biocalibrations were performed. Lights out for the participants occurred between 22:00–23:00, and they were allowed again to sleep for 8 uninterrupted hours before being awoken. During sleep, EEG data were monitored, and the closed-loop stimulation intervention was started when 4 min of continuous N2/N3 sleep was observed and was allowed to run through the remainder of the night. If the participant showed signs of waking, or needed to use the restroom, the stimulation was paused, and resumed after the participant was again in N2/N3 sleep. Upon waking, participants were allowed to use the restroom, and were offered water and snacks. They filled out the KSD to assess subjective sleep quality. Next, they completed two more test blocks of the target detection task (“morning test”) to assess the effect of SWO augmentation on consolidation/performance, filled out the strategy questionnaire, and then were disconnected from the EEG hardware, and released. Similar to the immediate test, each block in the morning test presented 60 images (30 repeated, 30 generalized). The repeated images used in morning test were different from those used in the immediate test.

For the day 2 follow-up, participants arrived approximately 24 h after their day 2 arrival (17:30), were prepped for EEG data collection, and were administered two more test blocks (“afternoon test”) to assess the effects of SWO augmentation on more long-term retention and performance. Note that each block of the immediate, morning, and afternoon tests presented 30 repeated and 30 generalized images, and there was no overlap in stimuli across these runs.

**FIGURE 2:**
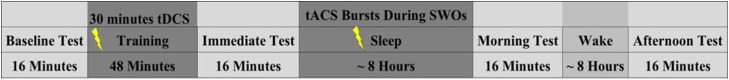
Experimental timeline for night 1 training/testing. Note night 2 training/testing was identical, but the stimulation condition (verum/sham) and the target category (people/object) were opposite of night 1. Nights 1 and 2 were separated by an average of 5.19 days (*SD* = 1.96 days).

**FIGURE 3:**
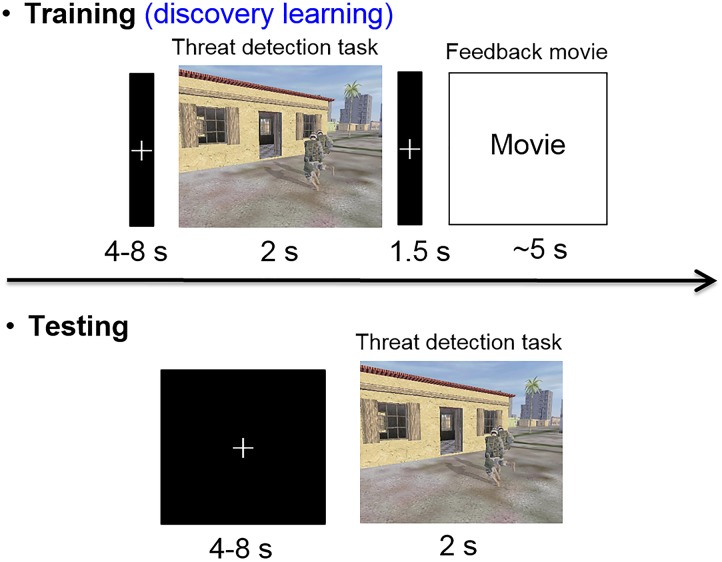
Timeline of training and test trials. Trials with targets present showed either object (e.g., trip wire, car bomb, barrel IED) or people (e.g., tank gunner, rock thrower, and rooftop sniper) targets during different training sessions. The response regarding the presence or absence of a target had to be made within the presentation duration (2 s) of each image. During a training trial with a target present, the feedback didn’t identify either the location or the identity of the target, so the participants had to discover the cues for the various targets through experience.

Approximately 5 days after completing the day 2 follow-up, participants came back to the laboratory for their night 2 and day 3 follow-up. The timeline and procedures were identical to night 1 and day 2 follow-up, the only differences being the target category (object targets/people targets) and stimulation condition (verum/sham) were opposite of their day 2 assignments. Upon completion of the day 3 follow-up, a final exit questionnaire was administered to gather subjective ratings from participants in terms of how they felt the intervention impacted their memory functioning generally. And they were debriefed, during which time they could ask questions about the nature of the experiment. See Figure 2 for a graphical description of the experimental procedures and Figure 3 for the target detection experimental procedure.

Sleep data were visualized with low and high pass filters at 0.5–35 Hz, with the exception of EMG, which was filtered between 10 and 100 Hz. Each 30-s epoch was visually inspected and assigned a stage of wake, NREM1, NREM2, NREM3/SWS, REM, or movement (Berry et al., 2012).

### Waking Electroencephalographic (EEG) Data Collection

A StarStim R32 simultaneous EEG/Stimulation device (Neuroelectrics, Inc.) was used. Participants were prepped and fitted with a neoprene EEG cap that incorporated 32 Ag-AgCl electrodes (solidgeltrodes: NE028, Neuroelectrics, Inc.), placed according to the extended 10–20 EEG system (P7, T7, CP5, FC5, F7, F3, C3, P3, FC1, CP1, Pz, PO4, O2, Oz, O1, PO3, CP2, Cz, FC2, Fz, AF3, Fp1, Fp2, AF4, P8, T8, CP6, FC6, F8, F4, C4, P4). Three of the channels were utilized for electrocardiogram (ECG) and electrooculogram (EOG) recordings: PO3 placed under the left collarbone for ECG, and AF3 and AF4 placed superior and lateral to the right outer canthus, and inferior and lateral to the left outer canthus, for vertical and horizontal EOG. Common Mode Signal (CMS) and Driving Right Leg (DRL) reference electrodes (stricktrodes: NE025, Neuroelectrics, Inc.) were placed on the preauricular, as stimulation was applied to the mastoids. Data was sampled at 500 Hz.

### Polysomnographic (PSG) Data Collection

For polysomnographic (PSG) data collection during sleep, the setup was nearly identical to wake, with a few exceptions. First, two EMG electrodes were placed on and under the chin in accordance with PSG recording guidelines set forth by the American Academy for Sleep Medicine (Berry et al., 2012) to help with sleep scoring. Second, EEG data were collected from 25 electrodes. For closed-loop SWO augmentation, four channels were dedicated for stimulation; namely, F3, F4, and bilateral mastoids (where T7, T8 electrodes were placed). Finally, as these channels were used for stimulation, they were omitted from EEG data collection. Of the remaining electrodes, Fp1 and Fp2 along with C3, C4, O1, and O2 were used for sleep staging.

### Waking Transcranial Direct Current Stimulation (tDCS)

Thirty minutes of continuous transcranial direct current stimulation (tDCS) was delivered during 48 min of training. A custom tDCS template for use during awake training was defined in the Neuroelectrics control software, CoreGUI. The anode electrode was set to +1000 μA, and the cathode was set to -1000 μA, for a total dose of 1000 μA (1.0 mA) for verum stimulation; for sham stimulation, the current values were +100 μA and -100 μA, for a total dose of 100 μA (0.1 mA). Two SPONSTIM 25 electrodes with saline soaked sponges (25 cm^2^) were affixed to the participants. For verum stimulation, the anode electrode was centered over the right sphenoid bone (electrode site F10), and the cathode electrode was placed on the upper contralateral arm. For sham stimulation, the placement, polarities, and duration were identical to those for verum stimulation, but the current was set to 0.1 mA instead of 1.0 mA.

Physical sensation ratings were solicited three times during tDCS administration: once after current ramp-up (approximately 1 min), 4 min following ramp-up before the first training run began (approximately 5 min after stimulation had begun), and immediately following the first training run (approximately 21 min after stimulation had begun). Participants were asked to rate three different types of sensations (itching, heat/burning, and tingling) on a 0–10 Likert scale, where 0 indicated no feeling of sensation at all and 10 indicated the worst possible feeling of sensation. Any report of a seven or above resulted in immediate cessation of stimulation and termination of the experiment, without penalty to the participant.

### Closed-Loop Transcranial Alternating Current Stimulation (CL-tACS) During Slow-Wave Oscillations

Our closed-loop algorithm for the electrical augmentation of memory consolidation first detected the presence of SWOs, which consist of slow synchronized positive and negative deflections of EEG that are associated with memory consolidation. The algorithm next attempted to match the stimulation frequency and phase with ongoing slow-wave activity such that maximal positive stimulation occurred at the UP states (positive half waves) of the endogenous SWOs, as prior work suggests that these are the periods during which coordinated memory replays between hippocampus and neocortex occur to facilitate long-term memory consolidation (Ji and Wilson, 2007). For robust SWO detection, a virtual channel was computed by averaging 13 fronto-central EEG channels (Cz, FC1, FC2, CP1, CP2, Fz, C4, Pz, C3, F3, F4, P3, P4 in the extended 10–20 system) to determine the overall synchronous activity of EEG recorded during sleep. The virtual channel allowed the observation of moments of relatively high slow-wave power, referred to as slow-wave events, while averaging out lesser magnitude activity on individual channels unrelated to the pattern of SWOs. The included channels were stored in a running 5-s buffer. They underwent moving average subtraction with a 1 s window (to center the mean of the signals at 0 μV). Noisy channels exceeding 500 μV min-to-max amplitude across the 5 s were rejected before the virtual channel was computed. Each discrete data fetch operation updated the buffer with a random transmission delay, which needed to be accounted for to plan and precisely time the next brain stimulation intervention.

**FIGURE 4:**
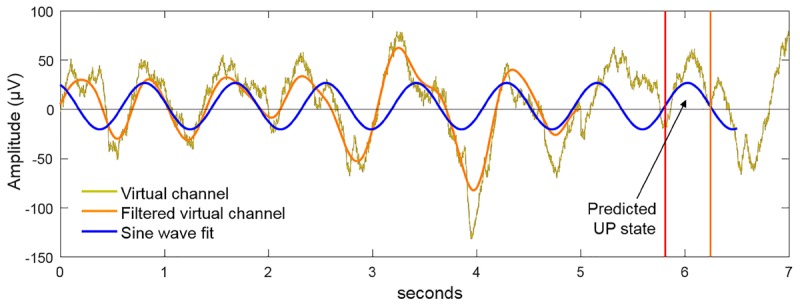
Method for closed-loop alignment of tACS to SWOs. The virtual EEG channel (gold) in the 5 s buffer is bandpass filtered in the SWO frequency range (0.5–1.2 Hz). If the relative power in the SWO band is >20% of the broadband power across 0.1–250 Hz, a sine wave at the dominant SWO frequency is fit to the filtered virtual channel and projected into the future to predict the time points of the next available UP states. By matching the phase of the tACS to this projected function, the dynamics of tACS and the predicted endogenous signal are aligned. For verum stimulation, 5 cycles of closed-loop tACS was applied in response to observed SWO events through the sleep.

The virtual channel data in the buffer was further processed to detect the presence of SWOs and predict the next UP state (see Figure 4). The power spectrum, computed by Fast Fourier Transform (FFT), was used to plan stimulation when the ratio of the cumulative power in the slow-wave band (0.5–1.2 Hz) was more than 20% of the total cumulative power from 0.1 to 250 Hz. If this SWO relative power threshold of 20% was crossed, the algorithm then filtered the data in the slow-wave band with a second-order zero-lag Butterworth filter. Next, a sine wave was fit to the filtered virtual channel using the identified dominant slow-wave frequency and by optimizing the amplitude, offset, and phase parameter values. The sine wave was then projected into the future, identifying the temporal targets that would synchronize brain stimulation to the predicted endogenous signal. Throughout this process, the dynamic latency associated with data processing was timed using the system clock. Together with distributions of calibrated latencies for data fetch and stimulation commands (mean = 5 ms, *SD* = 2 ms), which were measured offline, the algorithm determined the correct time point to communicate with the hardware to initiate the stimulation. For instance, suppose at a given moment the algorithm initiates data fetch to populate the buffer with the last 5 s of EEG data, the data becomes available for processing a few ms (say, 6 ms) into the future based on sampling from the distribution for data fetch latency. Then, say it takes 100 ms for data processing to predict the next UP state, which happens to be 600 ms into the future from the starting time point. If it takes a few ms (say, 7 ms) to physically initiate stimulation based on sampling from the distribution for stimulation command latency, the algorithm would wait 487 ms after the EEG processing step to send the stimulation command to the device. tACS was applied for 5 cycles at the detected SWO frequency. Should the next possible stimulation start time be later than the start of the next predicted UP state, yet at least 300 ms before its end, then synchronous stimulation was initiated and continued until 4 full cycles are completed (where a cycle is defined as the progression from 0° phase to 360° phase). In the event that at least 300 ms of UP state stimulation was not possible, then the algorithm planned the stimulation to start at the next upcoming UP state based on the continued sine wave projections from the buffer. Following the offset of tACS delivery, the system idled for 3 s to avoid the collection of stimulation artifacts in the data buffer, then resumed the cycle of data update in the buffer, data processing and predictions, and stimulation planning as the criteria specified above are met. Thus, our closed-loop system adapted and adjusted stimulation parameters online in order to ensure the proper administration of stimulation at the correct temporal targets to match the predicted transient brain states of interest. It is able to minimize the pitfalls of temporal inaccuracies that arise as a result of variable delays intrinsic to any recording/stimulation/processing hardware. On sham nights, UP states were similarly predicted but no stimulation (i.e., 0 mA) was applied. For stimulation on verum nights, 1.5 mA sinusoidal currents (peak-to-peak = 3 mA) were applied at F3, F4, T7, and T8 with pistim electrodes (NE024, Neuroelectrics, Inc.) using the method stated above with F3 and F4 in phase with each other and with ongoing SWOs, and 180° out of phase with T7 and T8. Note T7 and T8 electrodes were placed on bilateral mastoids.

### *Post hoc* Sleep EEG Analysis

Analysis of the sleep EEG data was performed using custom-built scripts implemented in Matlab R2016a (The MathWorks) taking advantage of various FieldTrip (Oostenveld et al., 2011) and EEGLab (Delorme and Makeig, 2004) functions. EEG data was extracted from verum night sessions and epoched into pre- and post-stimulation windows. Pre-stimulation epochs captured -6.4 to -0 s before tACS onset, and post-stimulation epochs captured 0–12.8 s relative to tACS offset. A segment-level artifact removal was done within each epoch by searching in 200 ms sliding windows for a peak-to-peak voltage change of 500 μV within each channel. Any segment that crossed this threshold was marked as bad and interpolated using non-artifact afflicted time points before and after the marked segments. Any channel that had more than 25% of its segments within a given epoch marked as bad was discarded, and the full epoch for that channel was interpolated using neighboring channels. After segment-level artifact removal, a pass of trial-level removal was done such that any channel that exceeded the 500 μV (min-to-max voltage change) threshold within a given tACS event was reconstructed by interpolation of its neighbors. Following artifact removal, all epochs were truncated to -6.4 to -1 s pre-tACS event and 3–12.8 s post-tACS event to ensure no stimulation artifacts lingered in the data. Finally, all epochs were mean centered, bandpass filtered between 0.1 and 125 Hz, bandstop filtered between 59 and 61 Hz, and all channels were re-referenced to the global average across channels.

### Spectral Power Methods

Time frequency decomposition was done in FieldTrip using Morlet wavelets. Before decomposition, symmetric (mirror) padding was used to extend the pre- and post-tACS event time-series to avoid edge artifacts in frequency decomposition. The series of wavelets used in the decomposition started with a width of 4 at the center frequency of 0.5 Hz, and subsequent center frequencies were chosen such that each wavelet was one standard deviation in the frequency domain from the previous wavelet. Simultaneously, the wavelet width was increased as a function of center frequency to minimize the combined uncertainty in time and frequency domains, with a starting width of 4 and a maximum width of 12. This yielded a time frequency representation with 52 approximately log spaced frequency bins from 0.5 to 100 Hz, and equally spaced time bins of 20 ms. Normalized power within each frequency bin was calculated by first *z*-scoring within each tACS event based on a mean and standard deviation in power estimated over the whole time period (-6.4 to 12.8 s). Relative power within each frequency bin was then calculated using a baseline period across tACS events by concatenating -3.5 to -3 s from all pre-tACS event periods and estimating a mean and standard deviation from this concatenated time series. These values were then used to *z*-score within frequency bins both the pre and post periods for each tACS event, to avoid single trial bias in spectral normalization (Ciuparu and Mureşan, 2016). This *z*-scored change in power was then averaged across the frequencies within the SWO band (0.5–1.2 Hz) for verum stimulation to yield a single channel × time × epoch matrix for each participant.

### Statistical Analyses

Data were analyzed within a repeated measures ANOVA framework, comparing “time” (within subjects: baseline, training, immediate, morning, afternoon), two image “types” when appropriate (within subjects: repeated images, generalized images), and two “stimulation” conditions (within subjects: verum, sham). Number of stimulation events for the verum night was entered into the model as a covariate for all analyses except the models that include pre-sleep immediate test performance. Verum night and sham night slow-wave events were highly correlated (Pearson’s *r* = -0.643, *p* = 0.005), and thus only verum event count was used as a covariate due to multicollinearity concerns, as well as the fact that it was the electrical augmentation that was of interest, not slow-wave events *per se*. Two participants were missing one time point of data each, and for these data points a Non-linear Iterative Partial Least Squares (NIPALS) algorithm (Wold, 1974) was run for data imputation in XLSTAT. For analysis of learning in the target detection task, two main outcome metrics were calculated: correct rate (hits plus correct rejections), and F1 score, which is the harmonic mean average of precision and recall (McSherry and Najork, 2008). Its range is from 0 to 1, where 1 is perfect precision and recall. F1 score is often used in the machine learning literature but can be applied to human cognition as well. Precision is calculated as the proportion of correct responses to all affirmative responses made [hits/(hits + false alarms)], and recall is calculated as the proportion of correct responses to all targets present [hits/(hits + misses)]. With these metrics, dependent variables were calculated using raw performance scores from baseline to immediate test (after waking tDCS), as well as morning and afternoon tests (after sleep intervention), and overnight performance changes from immediate to morning and afternoon tests for repeated and generalized images. Finally, contrast scores were calculated by subtracting repeated from generalized overnight performance changes for the morning and afternoon tests separately. Greenhouse-Geisser correction was used for interpretation of within subjects effects in the case of violated sphericity, and pairwise comparisons were adjusted using a Bonferroni correction. Curve fitting analyses were performed, fitting both linear and quadratic effects, in order to investigate dose-dependent effects of our intervention on post-sleep raw performance. For these analyses, two observations per subject were used (namely, verum event count with verum performance, and sham event count with sham performance). Since we are primarily interested in the effects of verum tACS on performance, and not necessarily the relationship between performance and slow-wave events *per se*, stimulation event counts for the sham night were set to 0 because no stimulation was applied. Data were analyzed with SPSS version 24 (Armonk, NY, United States: Ibm Corp, 2017). There were no significant differences between verum and sham nights in terms of the number of awakenings as determined by an experienced rater, suggesting that our CL-tACS intervention did not disturb sleep.

## Results

### Overall Raw Scores

**FIGURE 5:**
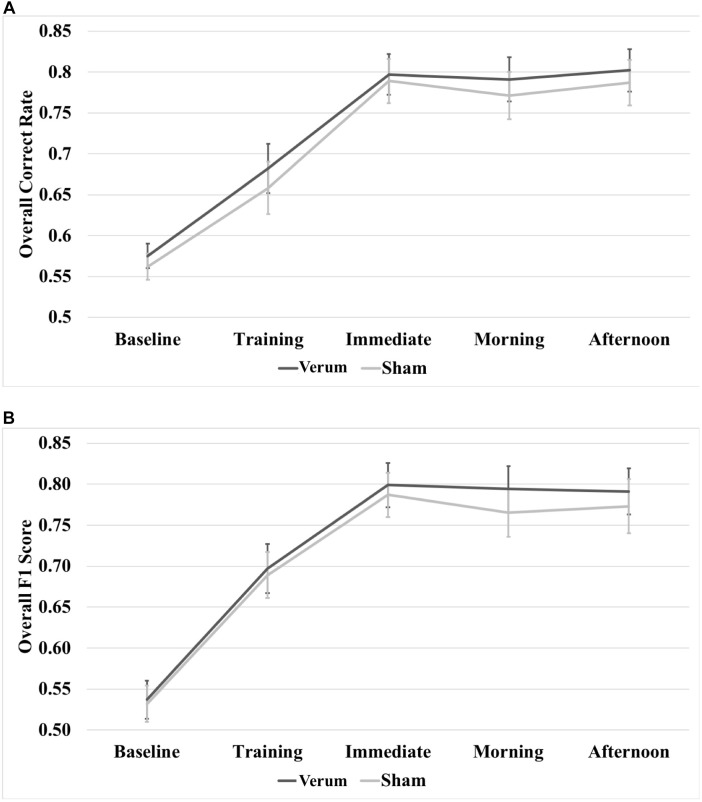
Raw performance scores through the experiment. **(A)** Overall correct rate through the experiment. **(B)** Overall F1 score through the experiment. There were no significant differences at any time point between verum and sham stimulation conditions. Error bars represent ±1 SEM.

A 5 × 2 (time^∗^stimulation) RMANOVA on raw overall correct scores revealed a main effect of time [*F*_(4,80)_ = 79.815, *p* < 0.000001], but no significant effect of stimulation or a significant interaction between time and stimulation (see Figure 5A). A 5 × 2 (time^∗^stimulation) RMANOVA on raw F1 scores also revealed a main effect of time [*F*_(4,80)_ = 71.868, *p* < 0.000001], but no significant effect of stimulation or a significant interaction between time and stimulation (see Figure 5B).

### Raw Scores (Immediate and Morning Tests)

**FIGURE 6:**
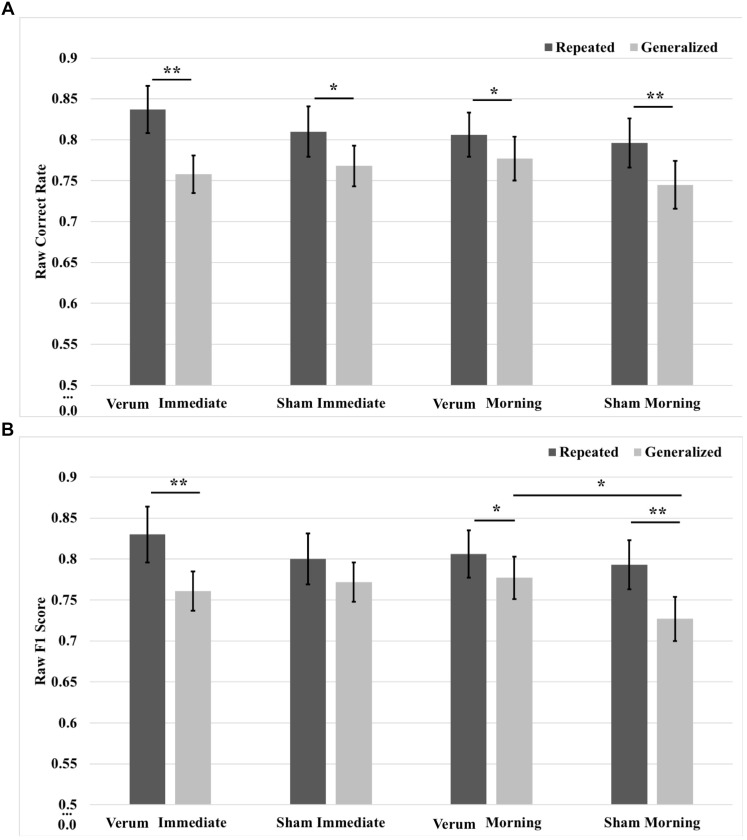
Raw performance scores from immediate (pre-sleep) and morning (post-sleep) tests broken down for repeated and generalized images in the verum and sham stimulation conditions. **(A)** Raw correct scores. Performance on repeated images was significantly higher than on generalized images for verum and sham stimulation at both the immediate and morning tests. **(B)** Raw F1 scores. There was a simple effect of image type for verum stimulation at the immediate test, with performance on repeated images better on generalized images. There was a trend for sham stimulation performing better than verum stimulation at the immediate test on generalized images; however, performance for sham stimulation on generalized images declined over sleep. Following sleep, the significant effect of image type remained for verum stimulation and was additionally seen for sham stimulation. There was a simple effect of stimulation condition at the morning test for generalized images, where verum stimulation led to improved performance compared to sham stimulation. Note that no significant effect was observed at the immediate test for tDCS applied during training for either image type. ^∗^*p* < 0.05, ^∗∗^*p* < 0.01, error bars represent ±1 SEM. Some of the immediate test data in **(B)** is also presented in Ketz et al. (2018).

In order to characterize the overnight performance changes and separate the effects of waking tDCS from sleep tACS, a 2 × 2 × 2 (time^∗^type^∗^stimulation) RMANOVA was run on raw correct rate and F1 scores, comparing performance at the immediate (after tDCS) and morning (after tACS) tests. Results for correct rate showed a three-way interaction of time^∗^type^∗^stimulation [*F*_(1,20)_ = 7.631, *p* = 0.0120], as well as a main effect of image type [*F*_(1,20)_ = 42.429, *p* = 0.000002], where performance for repeated images was greater than for generalized images by a mean marginal difference of 5.0%, collapsed across time and stimulation condition. Investigation of the interaction showed a simple effect of image type within levels of time and stimulation at the immediate test for both verum [*F*_(1,20)_ = 45.249, *p* = 0.000002] and sham [*F*_(1,20)_ = 5.285, *p* = 0.324] stimulation, as well as at the morning test for both verum [*F*_(1,20)_ = 4.670, *p* = 0.0429] and sham [*F*_(1,20)_ = 9.072, *p* = 0.0068] stimulation. Performance on repeated images was greater than for generalized images for either stimulation condition at both the immediate and morning tests (7.9, 4.1, 2.9, and 5.2%, respectively, for immediate verum/sham and morning verum/sham; Figure 6A). Note there were no significant differences in pre-sleep correct rate for either image type between verum and sham stimulation conditions.

Results for F1 score also showed a three-way interaction of time^∗^type^∗^stimulation [*F*_(1,20)_ = 12.761, *p* = 0.0019], as well as a main effect of image type [*F*_(1,20)_ = 26.481, *p* = 0.000049], where performance for repeated images was greater than for generalized images by a mean marginal difference of 4.8%, collapsed across time and stimulation condition. Investigation of the interaction showed a simple effect of image type within levels of time and stimulation at the immediate test for verum stimulation [*F*_(1,20)_ = 14.083, *p* = 0.0012], as well as at the morning test for both verum [*F*_(1,20)_ = 5.194, *p* = 0.034] and sham [*F*_(1,20)_ = 18.168, *p* = 0.00038] stimulation. Generalized image performance was greater for verum stimulation compared to sham stimulation by 5.0% at the morning test (*p* = 0.010). Performance on repeated images was greater that generalized images for either stimulation condition at both the immediate and morning tests (6.8, 2.9, and 6.6%, respectively, for immediate verum and morning verum/sham; Figure 6B). For F1 score metric as well, there were no significant pre-sleep differences for either image type between verum and sham stimulation conditions. In other words, waking tDCS did not have any learning effects in pre-sleep performance.

### Repeated/Generalized Overnight Changes

**FIGURE 7:**
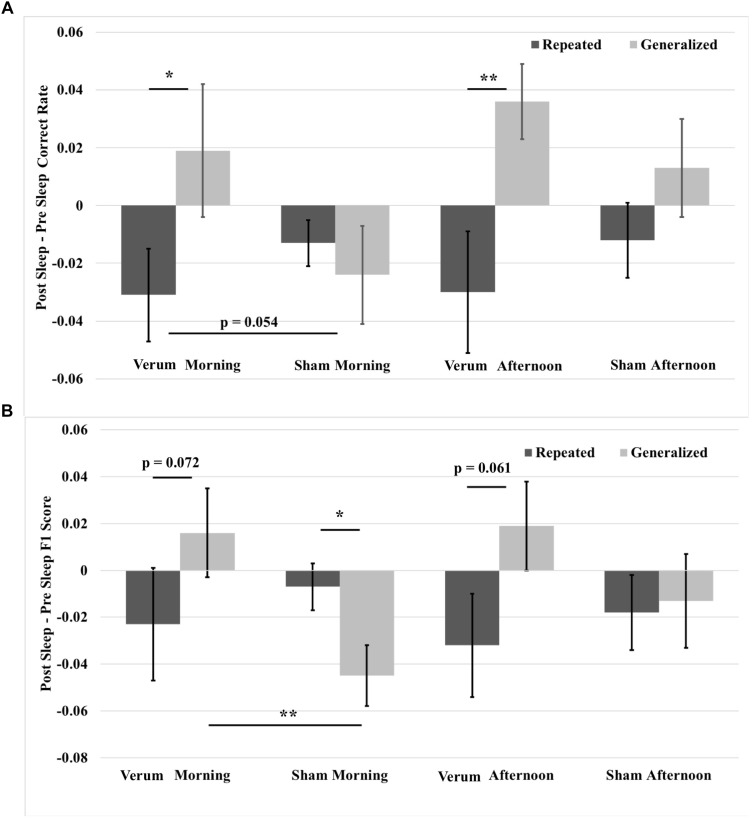
Overnight performance changes from immediate to morning and afternoon tests broken down for repeated and generalized images in the verum and sham stimulation conditions. **(A)** Overnight correct rate changes. The overnight correct rate change on generalized images was greater than on repeated images for verum stimulation at both morning and afternoon tests. Further, the overnight correct rate change on generalized images at the morning test for verum stimulation was greater than that for sham stimulation at a trend level. **(B)** Overnight F1 score changes. The overnight F1 score change for generalized images was significantly greater for verum stimulation compared to sham stimulation at the morning test. Further, the overnight F1 score change on generalized images for verum stimulation was greater than on repeated images at both morning and afternoon tests at a trend level. However, the overnight F1 score change on repeated images for sham stimulation was significantly greater than on repeated images at the morning test. Some of the data in **(B)** is also presented in Ketz et al. (2018), and is further broken down by morning and afternoon tests. ^∗^*p* < 0.05, ^∗∗^*p* < 0.01, error bars represent ± SEM.

A 2 × 2 × 2 (time^∗^stimulation^∗^type) RMANCOVA with tACS count as a covariate on correct rate overnight changes from immediate to morning and afternoon tests revealed a marginal effect of the covariate (namely, verum stimulation count) on performance [*F*_(1,19)_ = 3.866, *p* = 0.064], and a main effect of image type [*F*_(1,19)_ = 11.132, *p* = 0.0034], where performance for generalized images was greater than for repeated images by a mean marginal difference of 3.3%. Investigation of simple effects of image type within levels of time and stimulation condition revealed significant effects for verum stimulation, where overnight performance change on generalized images was greater than that on repeated images at both the morning [*F*_(1,19)_ = 7.504, *p* = 0.0099] and afternoon [*F*_(1,19)_ = 9.702, *p* = 0.018] tests by 5 and 6.6%, respectively. No significant effects were found for sham stimulation. See Figure 7A for these results.

A 2 × 2 × 2 (time^∗^stimulation^∗^type) RMANCOVA on F1 score overnight changes from immediate to morning and afternoon tests revealed a main effect of image type [*F*_(1,19)_ = 5.7679, *p* = 0.0265], where performance for generalized images was greater than for repeated images by a mean marginal difference of 1.5%, and a three-way interaction of time^∗^stimulation^∗^type [*F*_(1,19)_ = 4.468, *p* = 0.0475]. Investigation of the three-way interaction revealed a simple effect of stimulation condition at the morning test for generalized images [*F*_(1,19)_ = 10.441, *p* = 0.0043], where verum stimulation led to a higher overnight change in F1 score than sham stimulation by 6.1%. No significant effects were found at the afternoon test, or at either time point for repeated images, suggesting that the effect of improved performance on generalized images did not come at the expense of impaired performance on repeated images. Investigation of simple effects of image type within levels of time and stimulation condition revealed marginal effects for verum stimulation at both morning [*F*_(1,19)_ = 3.625, *p* = 0.0721] and afternoon [*F*_(1,19)_ = 3.960, *p* = 0.061] tests, where overnight performance change for generalized images was greater by 4.0 and 5.1%, respectively, than for repeated images. A simple effect was found at the morning test for sham stimulation [*F*_(1,19)_ = 6.188, *p* = 0.0229], where overnight performance change for repeated images was greater than for generalized images by 3.7%. See Figure 7B for these results.

### Contrast in Generalized vs. Repeated Overnight Changes

**FIGURE 8:**
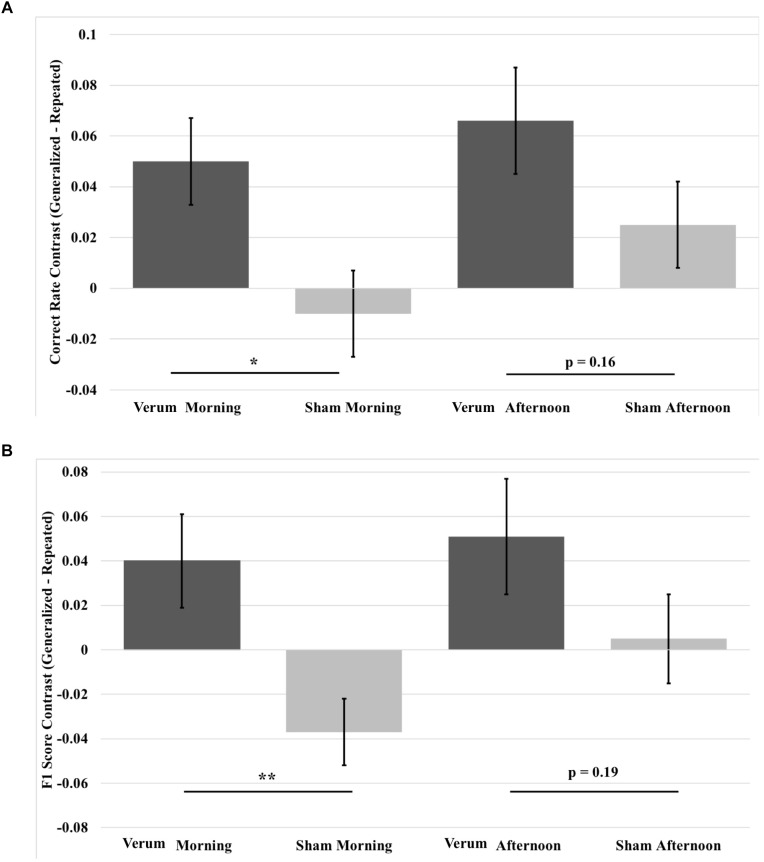
Contrast in overnight performance changes between generalized and repeated images from immediate to morning and afternoon tests in the verum and sham stimulation conditions. **(A)** Generalized vs. repeated image contrast in overnight correct rate changes. The overnight correct rate change for generalized images over that for repeated images was significantly greater for verum stimulation compared to sham stimulation at the morning test. This difference was at a trend level at the afternoon test. **(B)** Generalized vs. repeated image contrast in overnight F1 score changes. The overnight F1 score change for generalized images over that for repeated images was significantly greater for verum stimulation compared to sham stimulation at the morning test. This difference was at a trend level at the afternoon test. ^∗^*p* < 0.05, ^∗∗^*p* < 0.01, error bars represent ±1 SEM.

Given the benefit of tACS on overnight performance change for generalized images compared to repeated images, we analyzed contrast scores for both correct rate and F1 score, which were obtained by subtracting repeated from generalized overnight performance changes for the morning and afternoon tests, using a 2 × 2 (time^∗^stimulation) RMANCOVA with tACS count as a covariate. Results for correct rate showed a marginal effect of verum stimulation count on performance [*F*_(1,19)_ = 3.927, *p* = 0.0621] and an effect of stimulation condition [*F*_(1,19)_ = 5.946, *p* = 0.0147], where verum stimulation performance was greater than sham by a mean marginal difference of 5.1%, which was driven by a simple effect of stimulation condition at the morning test [*F*_(1,19)_ = 7.261, *p* = 0.0143], where verum stimulation performance was greater than sham by 6% (see Figure 8A).

Results for F1 score revealed a main effect of stimulation condition [*F*_(1,19)_ = 7.195, *p* = 0.0247], an interaction of time^∗^stimulation [*F*_(1,19)_ = 4.486, *p* = 0.0475], and a main effect of verum stimulation count [*F*_(1,19)_ = 4.591, *p* = 0.0453]. Investigation of the interaction showed a simple effect of stimulation condition at the morning test [*F*_(1,19)_ = 12.196, *p* = 0.0024], where the generalized vs. repeated contrast in overnight F1 score change for verum stimulation was greater than for sham stimulation by a mean marginal difference of 7.7%. No significant effect was found at the afternoon test (see Figure 8B).

### tACS Dose Effects

**FIGURE 9:**
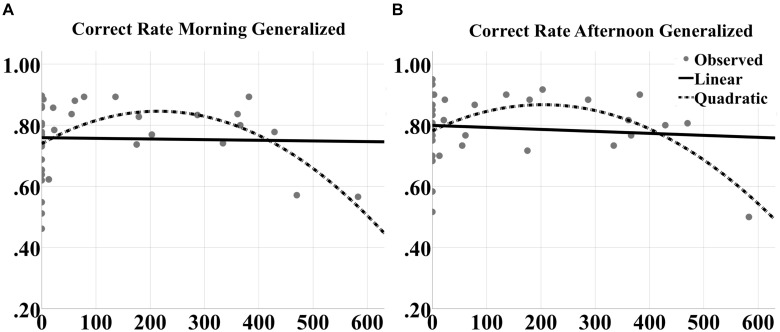
Inverted U-shaped dose effects for correct rate metric. Linear and quadratic fits to raw correct rate performance on generalized images as a function of number of tACS events during either experimental night are shown for the **(A)** morning and **(B)** afternoon tests separately. The legend is in **(B)**. The quadratic fits in each panel were significant.

To better understand the contribution of closed-loop tACS to sleep-dependent memory generalization, we analyzed dose effects of tACS event count through the night on raw performance in correct rate and F1 score at morning and afternoon tests for both generalized and repeated images separately, as well as the contrast in generalized vs. repeated overnight performance changes, across participants and between the two experimental nights. One subject was an outlier (>2 standard deviations from the mean) for verum stimulation count, and one subject was an outlier for behavioral performance. They were thus excluded from the curve fitting analyses. Linear and quadratic trends were fit for each outcome variable. There were no significant linear effects for any measure. However, quadratic effects (in the shape of an inverted U) were observed for correct rate measures at both morning [*F*_(2,39)_ = 4.472, *p* = 0.0182; quadratic *t* = -2.984, *p* = 0.005, *R*^2^ = 0.195; Figure 9A] and afternoon [*F*_(2,39)_ = 4.187, *p* = 0.0229; quadratic *t* = -2.820, *p* = 0.0076, *R*^2^ = 0.185; Figure 9B] tests for generalized images. But no significant effects were obtained for repeated images or the contrast scores.

**FIGURE 10:**
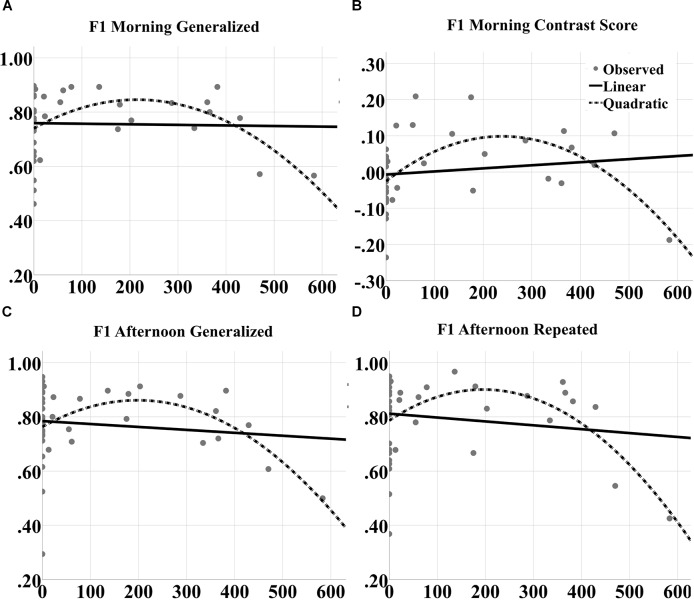
Inverted U-shaped dose effects for F1 score metric. Linear and quadratic fits to raw F1 score performance on generalized images as a function of number of tACS events during either experimental night are shown for the **(A)** morning and **(C)** afternoon tests separately. The legend is in **(B)**. Further, the fits to raw F1 score on repeated images for the afternoon test are shown in **(D)**. And the fits to the contrast in overnight F1 score changes for generalized vs. repeated images at the morning test are shown in **(B)**. The quadratic fits in each panel were significant.

For F1 scores, significant quadratic effects were also observed at both morning [*F*_(2,39)_ = 5.191, *p* = 0.0102; quadratic *t* = -3.042, *p* = 0.0043, *R*^2^ = 0.195; Figure 10A] and afternoon [*F*_(2,39)_ = 4.625, *p* = 0.0161; quadratic *t* = -2.901, *p* = 0.0062, *R*^2^ = 0.200; Figure 10C] tests for generalized images. Furthermore, significant quadratic effects were obtained for repeated images at the afternoon test [*F*_(2,39)_ = 5.191, *p* = 0.0102; quadratic *t* = -3.042, *p* = 0.0043, *R*^2^ = 0.219; Figure 10D] and for the contrast score (generalized – repeated overnight change) at the morning test [*F*_(2,39)_ = 7.563, *p* = 0.0017; quadratic *t* = -3.739, *p* = 0.00062, *R*^2^ = 0.290; Figure 10B].

**FIGURE 11:**
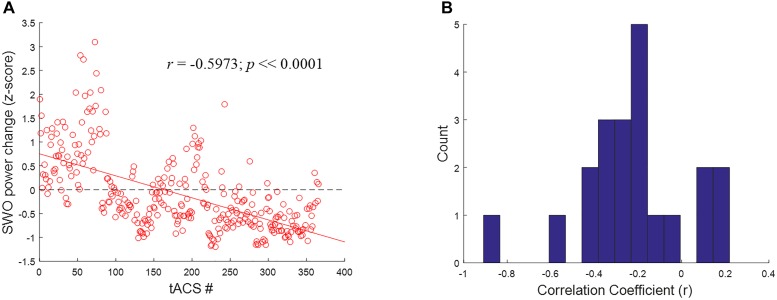
Closed-loop tACS causes early facilitation as well as later suppression of SWOs. **(A)** For a representative subject, relative SWO power change (*z*-score) induced by closed-loop tACS as a function of the cumulative number of tACS events through the night. The red solid line shows the linear fit to the data, while the black dashed line signifies the baseline condition (i.e., no modulation) to highlight the earlier facilitation and the later suppression of post-stimulation SWO power. **(B)** Histograms of correlation coefficients (*r*) across the participants, with 15 bins, for the linear relationship illustrated in **(A)**.

To better understand the mechanisms underlying the inverted U-shaped dose effects seen in Figures 9, 10, we analyzed the tACS-induced EEG spectral power changes for SWO band (0.5–1.2 Hz) from pre- to post-stimulation as a function of stimulation event through the verum nights. For each stimulation event, the *z*-scored changes in SWO spectral power were computed over the post-stimulation epoch of 3–10 s (relative to tACS offset) and averaged across all EEG channels. This analysis was motivated by the refractory dynamics seen in post-stimulation EEG SWO power relative to pre-stimulation baselines when averaged across the night (Ketz et al., 2018). As shown in Figure 11A for a representative participant, we found that the linear relationship between the tACS-induced local change in SWO power and the cumulative number of tACS events through the night was negative for about 81% of the participants (17 of 21). In fact, the mean of the distribution of correlation coefficients (*r*) across the participants was significantly different than zero (mean = -0.2192, *t* = -3.9012, *p* < 0.001, two-tailed one-sample *t*-test; Figure 11B). Furthermore, 52.38% of the participants (11 of 21) demonstrated an explicit flip in their linear fits from post-stimulation enhancement of SWO power during initial tACS applications to post-stimulation suppression of SWO power during later tACS applications through the night. Results presented in Figure 11 are consistent with significantly fewer number of slow-wave events during verum nights, likely due to tACS-induced refractory dynamics and possibly the loss of some data due to EEG noise caused by tACS, compared to sham nights (Ketz et al., 2018).

## Discussion

In this study and Ketz et al. (2018), we sought to improve sleep-dependent memory consolidation processes through closed-loop tACS delivered during SWOs while participants slept in the laboratory. Our algorithm predicted UP states of SWOs and delivered transient tACS at the same frequency and in phase with these endogenous oscillations. Ketz et al. (2018) showed that our closed-loop tACS was primarily applied during NREM stages 2 and 3 compared to the other sleep stages (see their Figure 3), and also validated the starting phase of predicted UP states with respect to ongoing SWOs using a *V*-test for circular uniformity based on artifact-free data from the sham nights (see their Figure 2). In this article we demonstrate a selective dose-dependent enhancement in memory performance for generalized images compared to repeated images in a target detection task whose learning is parahippocampally-mediated (Clark et al., 2012). In particular, we show an inverted U-shaped dose effect of closed-loop tACS on post-sleep generalization performance in the target detection task, which is interpreted in terms of tACS-induced faciliatory and subsequent refractory dynamics of self-regulating SWOs in scalp EEG. Our intervention could be enhancing the brain’s natural processes during sleep that integrate information into long-term memory for improved generalization after sleep. The approach is flexible enough to deliver stimulation in concert with other neural oscillations (such as spindles and ripples) during both sleep and wake.

This work is seminal in terms of employing closed-loop non-invasive electrical stimulation to boost hippocampally-dependent memory processes occurring during sleep, as all previous studies of electrical stimulation targeting SWOs have only used open-loop methods to deliver stimulation. The phase and frequency of the applied stimulation is extracted from ongoing brain activity, providing the potential for entraining brain oscillations as well as enhancing their synchrony across brain areas, giving our intervention an advantage over previous work. A recent feedback-controlled method (Lustenberger et al., 2016), applying transient 12 Hz tACS in response to spindle activity during sleep, modulated overnight change in performance on a procedural memory task (namely, sequence tapping) but not on a declarative memory task (namely, word pairs). We are the first to not only adapt both phase and frequency of tACS in closed loop during sleep, but also enhance memory generalization performance in a discovery learning paradigm.

Previous work has shown that 30 min of 2.0 mA tDCS applied over right inferior frontal gyrus during training leads to a doubling in performance improvement, measured as the change in performance from baseline to immediate tests, for the target detection task used in the current study (Clark et al., 2012). This effect is one of the few cognitive tDCS studies to be replicated independently (Falcone et al., 2012). Coffman et al. (2012) investigated the interactions of tDCS and stimulus characteristics and found that verum tDCS led to a greater improvement in change from baseline to immediate tests for repeated compared to generalized images prior to sleep, likely enhancing veridical recall. By contrast, the current results showed the opposite pattern, with a larger effect of closed-loop sleep tACS on generalized compared to repeated images. Thus, there appears to be a differing effect when attempting to optimize performance on this task by combining waking tDCS and closed-loop sleep tACS, which likely depends on when and how the brain stimulation is applied. Further, there was no significant effect of waking tDCS on any metric in the current study prior to sleep, likely because 1.0 mA of current was delivered here, which was chosen in part to reduce the amount of stimulation (current × duration) for each individual participant over the course of the experiment, as opposed to 2.0 mA of current used in our previous tDCS-only awake studies. Note that the performance improvements on generalized images for verum stimulation did not come at the expense of repeated image performance. In fact, raw performance on repeated images compared to generalized images was greater for both stimulation conditions at the pre-sleep immediate test as well as at the morning test. However, after sleep, generalized image performance was increased for verum CL-tACS, but not for sham stimulation.

A limitation of the current study is the confounding factor of waking tDCS for the verum stimulation condition in accounting for the significant overnight performance changes for generalized images. In other words, we have not directly dissociated the individual contributions of waking tDCS and sleep tACS to overnight performance changes. However, previous work did not find any overnight improvements in target detection following waking tDCS alone over 24 h (Falcone et al., 2012). Further, as noted above, a more effective dose (2 mA) of tDCS during training had been shown to improve performance for repeated images significantly more than for generalized images before sleep (Coffman et al., 2012). Moreover, the current study shows significant inverted U-shaped dose effects of sleep tACS for post-sleep performances on generalized images in the context of a fixed waking tDCS dose of 1 mA over 30 min. These observations provide indirect evidence for the unique contribution of the closed-loop tACS in boosting long-term memory generalization for the target detection task.

We propose that the 5-cycle bursts of our closed-loop tACS transiently enhance the power of SWOs through the night, which greatly boosts the transfer and consolidation of recently acquired task information from short-term storage in hippocampus to long-term storage in neocortical areas (see “Introduction” section). In support of this possibility, Ketz et al. (2018) found significant tACS-induced changes in EEG spectral power for SWO band within the observable post-stimulation period (3–10 s from tACS offset) using a spatiotemporal clustering algorithm. Note the changes during the actual tACS application (5 cycles: 4.17–10 s) and the immediate 3 s following tACS could not be analyzed because of stimulation artifacts. Compared to sham stimulation, our closed-loop tACS was shown to enhance (from 3.02 to 4.24 s following tACS offset) as well as subsequently impair (from 4.28 s to at least 10 s following tACS offset) the power of SWOs, which in turn showed increased SWO-spindle coupling. Furthermore, both of these positive and negative modulations in SWO power were significantly correlated with the overnight F1 score change for generalized images, as well as the contrast in generalized vs. repeated overnight F1 score change, across subsets of clustered EEG channels in certain post-stimulation epochs.

Inverted U-shaped dose effects for tACS, presented in Figures 9, 10, point to a possible optimal number of stimulation events throughout the night in order to produce a benefit in performance of this task. If too few stimulations are applied, performance does not appear to be improved; likewise beyond a certain dose, continued application of closed-loop tACS may perpetuate and even extend the compensatory refraction in response to significant enhancements in SWO power as a result of initial applications earlier in the night (Figure 11). Prolonged suppressions of SWOs may have a negative impact on sleep consolidation that would have occurred earlier, such that the influence of dose is actually an inverted U curve. The optimal dose for a given individual can be tracked online during the night by monitoring for the occurrences of stimulation-induced suppression in SWO power following each tACS event.

To further understand and validate the mechanisms of the phenomenon under consideration, it would be necessary to accomplish both enhancement and impairment of memory consolidation for different suboptimal and optimal doses. Closed-loop tACS has the capability to both enhance endogenous brain rhythms (by stimulating in phase and frequency) and disrupt them (by stimulating out of phase and frequency), so theoretically it would be possible to improve or impair consolidation. Future studies should include this manipulation by either stimulating out of phase or at a different frequency to impair memory consolidation.

Sleep research presents a host of unique problems with data collection, including participant comfort and attrition (in the case of multi-night studies like the current design). Our intervention, while effective in modulating memory consolidation, was the first of its kind, which necessitated finding solutions to novel problems. The StarStim R32 EEG/stimulation device is relatively new and was not specifically designed for sleep research. Sleep EEG systems are designed to be comfortable enough to allow hours of sleeping on a pillow, and robust enough for reliable operation when connected to a tossing participant. We were the first group to attempt to use the R32 for closed-loop stimulation during wake as well as during sleep, which produced a host of technical hurdles that had to be overcome, in terms of being able to fetch EEG data in real time, keeping the electrode cap affixed to the participant throughout the night, and ensuring the continual operation of the device. One limitation of electrical stimulation during sleep, as in wake, is that a small subset of participants cannot tolerate the physical sensations associated with brain stimulation. Thus, it could be argued that electrical stimulation is suboptimal to auditory stimulation, however, the incidence of intolerability for electrical stimulation is very low on average, and electrical stimulation is robust against interference from ambient sensory stimuli in the environment owing to its non-sensory nature. The latter is likely a potential problem for auditory interventions for memory enhancement applications in less-than-controlled, real-world settings.

Though we used a task that was shown previously to be sensitive to tDCS (Clark et al., 2012), the within subjects counterbalanced experimental design required that the task be modified such that it could be delivered over the course of two experimental days. The choice to split the stimulus set based on target characteristics, creating an object set and a people set of stimuli, may have rendered the task too easy to learn, as participants could extrapolate from one stimulus set to the other when learning for night 2. Though the manipulation order was counterbalanced across the participants reported in this study, this carryover effect makes it harder to find a behavioral effect of our closed-loop tACS intervention. Despite this, it is notable that we found a significant improvement in overnight performance change for generalized compared to repeated images between the verum and sham stimulation conditions, with an effect size comparable to those reported previously in the literature. In particular, Marshall et al. (2006) demonstrated a 3.85% improvement in post-sleep performance of word recall with verum stimulation compared to sham based on 46 word-pairs, which is comparable to the current study where verum stimulation outperformed sham by 5% for F1 score on generalized images at the morning test.

This study has focused on declarative memory consolidation, in the context of improving the skill of target detection in static images. Future studies utilizing closed-loop tACS could be conducted with a more traditional assay of declarative memory consolidation. Paired associates is a foundational task used in most of the previous electrically augmented sleep-dependent memory consolidation studies, which have delivered stimulation in an open-loop manner only. Investigating the effects of closed-loop tACS on a paired associates task can provide a direct comparison with prior open-loop approaches (Marshall et al., 2006, 2011; Sahlem et al., 2015).

Our CL-tACS intervention can be optimized by personalizing the most critical parameter of the SWO relative power threshold using prior sleep data from a given participant. The participant’s whole-night polysomnographic recordings can be staged by an expert rater, in order to extract the distribution of relative power of the SWO band in the identified NREM sleep stage 3 (N3), when SWOs are most likely to occur (Rasch and Born, 2013). SWO relative power threshold for triggering CL-tACS can then be set to the median of this distribution. Further, the ongoing SWOs during sleep can be highly variable and drift in frequency and amplitude over time. As a result, UP state predictions derived using data collected further in the past could be less reliable. For this reason, the buffer length could be shortened to be just long enough to contain one or two cycles of SWOs at the lower bound frequency of 0.5 Hz (2 or 4 s). Increasing or personalizing the SWO relative power threshold and shortening the buffer length will certainly minimize any false predictions of UP states, such as those triggered in sleep stages other than N2 and N3 as well as those not coinciding with actual UP states, to trigger the CL-tACS intervention. The number of cycles and the number of applications of CL-tACS can be adjusted dynamically through the night based on tracking the EEG biomarkers of memory consolidation in the post-stimulation periods (Ketz et al., 2018). In other words, CL-tACS can be adapted gradually to maximize the post-stimulation biomarkers. It could also be the case that the optimal time for enhancing memory consolidation and generalization is during the first two sleep cycles. Several studies show an improvement in learning with an intervention over the course of a nap (e.g., Rudoy et al., 2009; Lewis and Durrant, 2011; Ladenenbauer et al., 2016), and perhaps our intervention would be more effective if we restricted its delivery to the first 3 h of sleep, when SWOs are the richest.

## Conclusion

This study shows that memory consolidation can be enhanced during sleep by applying closed-loop tES matched to the endogenous phase and frequency of SWOs in individual participants. The capability that we have developed here to estimate in real time the phase and frequency of endogenous brain activity and use that information to control non-invasive brain stimulation in closed loop during sleep holds tremendous potential. Sleep is fundamentally important to human health and wellbeing, and understanding it more fully could lead to a host of benefits. tES is a safe, low-risk, cost-effective tool that can be used to not only understand but also augment sleep-dependent memory consolidation processes. In future studies, closed-loop tES might be useful for a variety of applications, such as accelerating skill acquisition and boosting task performance, optimizing sleep quality and ameliorating cognitive symptoms associated with sleep disorders and other disorders that involve sleep such as dementia, schizophrenia, chronic pain, and many others. Preserving sleep quality during the application of electrical stimulation overnight is crucial given the myriad benefits a restful night of sleep has on overall health. Future studies will investigate the effects of CL-tACS on subjective sleep quality. The closed-loop tES methods developed here may one day help to improve cognitive performance and overall quality of life.

## Author Contributions

AJ, NB, VC, and PP conceived and designed the study. JC and PP developed the closed-loop tACS method. AJ, NB, CR, AC, ML, BR, MeH, and HG collected data for the study. AJ organized the database. AJ, SS, and PP performed the statistical analyses. AJ wrote the first draft of the manuscript. JC, NB, CR, NK, BR, HG, MiH, VC, and PP wrote sections of the manuscript. All authors read and revised the manuscript, and approved the submitted version.

## Conflict of Interest Statement

JC, NK, SS, MiH, and PP were employed by HRL Laboratories, LLC, JC, and PP have a pending patent application on the closed-loop tACS method. The remaining authors declare that the research was conducted in the absence of any commercial or financial relationships that could be construed as a potential conflict of interest.
